# Efficiency and bacterial diversity of an improved anaerobic baffled reactor for the remediation of wastewater from alkaline-surfactant-polymer (ASP) flooding technology

**DOI:** 10.1371/journal.pone.0261458

**Published:** 2022-01-07

**Authors:** Dong Wei, Xinxin Zhang, Chunying Li, Min Zhao, Li Wei

**Affiliations:** 1 School of Life Sciences, Northeast Forestry University, Harbin, Heilongjiang, People’s Republic of China; 2 State Key Laboratory of Urban Water Resource and Environment, Harbin Institute of Technology, Harbin, Heilongjiang, People’s Republic of China; 3 Guangzhou HKUST Fok Ying Tung Research Institute, Guang zhou, Guangdong, People’s Republic of China; 4 School of Energy and Civil Engineering, Harbin University of Commerce, Harbin, Heilongjiang, People’s Republic of China; South China University of Technology, CHINA

## Abstract

Alkaline-surfactant-polymer (ASP) flooding technology is used to maximize crude oil recovery. However, the extensive use of alkaline materials makes it difficult to treat the water used. Here, an improved multi-zone anaerobic baffled reactor (ABR) using FeSO_4_ as electron acceptor was employed to treat the wastewater from ASP flooding technology, and the effects on major pollutants (hydrolyzed polyacrylamide, petroleum substances, surfactants suspended solids) and associated parameters (chemical oxygen demand, viscosity) were evaluated. Gas chromatography-mass spectrometry (GC-MS) was used to follow the degradation and evolution of organic compounds while high-throughput DNA sequencing was used to determine the bacterial diversity in the ABR. The results obtained after 90 d of operation showed decreases in all parameters measured and the highest mean removal rates were obtained for petroleum substances (98.8%) and suspended solids (77.0%). Amounts of petroleum substances in the ABR effluent could meet the requirements of a national standard for oilfield reinjection water. GC-MS analysis showed that a wide range of chemicals (e.g. aromatic hydrocarbons, esters, alcohols, ketones) could be sequentially removed from the influent by each zone of ABR. The high-throughput DNA sequencing showed that the bacteria *Micropruina*, *Saccharibacteria* and *Synergistaceae* were involved in the degradation of pollutants in the anaerobic and anoxic reaction zones, while *Rhodobacteraceae* and *Aliihoeflea* were the main functional microorganisms in the aerobic reaction zones. The results demonstrated that the improved ABR reactor had the potential for the treatment of wastewater from ASP flooding technology.

## Introduction

Alkaline-surfactant-polymer (ASP) flooding is an efficient tertiary recovery technique used for the extraction of crude oil which cannot be recovered during the second stage [1]. ASP flooding technology uses alkali, surfactant and polymer agents to displace crude oil [2], and the mean recovery is significantly improved compared with the water flooding method [3]. ASP flooding technology has been applied in China, India, Russia, Canada and other countries [4]. Although ASP flooding technology has greatly improved the recovery rate of crude oil, remediation of the process water is an inevitable issue [5,6] limiting further development of the technology.

The composition of the ASP flooding process water is complex since the use of alkali, surfactants and polymers results in many pollutants including polymers and surfactants. Inevitably, separation of the emulsion resulting from water-polymer-surfactant interactions is challenging and increases the difficulty of oilfield wastewater treatment. The relatively high content of petroleum hydrocarbons in contaminated process water also represents a significant environmental hazard [7]. The Daqing oilfield in Heilongjiang province is the largest oilfield in China where ASP flooding technology is widely used in the recovery of crude oil. Here, treated wastewater from ASP flooding is reinjected into the oil reservoir to maintain its pressure. The reinjected water is required to meet a national standard for the use of reinjection water for high permeability reservoirs with oil and suspended solids contents of < 20 mg/L each (Oil and gas development Standardization Technical Committee, 2012).

To date, the major physical and chemical methods developed and applied to treat ASP flooding wastewater include membrane distillation [8,9], membrane filtration [10,11], air flotation [12], electrocoagulation [13], and adsorption [14]. However, the high operating and maintenance costs are the shortcomings of these technologies, which have a negative impact on their overall performance and limit the expanded application of these technologies [15].

Microorganisms play an important role in environmental treatment and restoration because of their ability to degrade pollutants [16]. Microorganisms can use hydrocarbons in oil as energy sources, and biological treatment may be an economic and effective method for the remediation of hydrocarbon-rich wastewater [17]. Several biological methods have been applied to oilfield wastewater treatment such as contact oxidation systems [18], membrane bioreactors [19] and fluidized bed bioreactors [20]. Previous studies have shown that petroleum hydrocarbons can be degraded by sulfate reduction [21–23], and the rate of degradation was sulfate dose-dependent [24]. Ferrous ion is a good additive for water bioremediation [25]. Previous studies have shown that the ferrous ion can reduce the adaptation period of sulfate-reducing bacteria and accelerate the consumption of H^+^ and sulfate via increasing hydrogenase activity, thus promoting the metabolic activity of the bacteria [26].

As the Daqing Oilfield enters the middle-late stage of crude oil extraction, ASP flooding technology is widely used to maximize the oil recovery. Consequently, large bodies of ASP flooding process water containing residual polymers, surfactants and inorganic salts have been generated. At present, the biological treatment of ternary produced water has not been involved by many researchers. We undertook the experiment to support the hypothesis that sulfur electron acceptor enhanced biological treatment of ASP flooding produced water. In this work, an anaerobic baffled reactor (ABR) was modified to treat ASP flooding process water obtained from the Daqing Oilfield. The active biomass (sludge) in conventional ABR reactors was replaced with polyurethane packing materials. The ABR device was divided into anaerobic, anoxic and aerobic reaction zones and FeSO_4_ was selected as the electron acceptor to improve the biological treatment efficiency. Since excess FeSO_4_ could combine with sulfur in the waste and form iron sulfide (precipitate), the amount of FeSO_4_ (as SO_4_^2-^) was maintained at 500 mg/L in the influent. A 90 d test was carried out in the laboratory and the main pollutants (hydrolyzed polyacrylamide (HPAM), petroleum substances, surfactants, suspended solids) and other key parameters (viscosity and chemical oxygen demand (COD)) were measured in the ABR treated process water to evaluate the biodegradation efficiency of the modified ABR system. The degradation of organic chemical pollutants in the ABR was followed by the analysis of the wastewater in different regions of the ABR using combined gas chromatography-mass spectrometry (GC-MS). High throughput DNA sequencing technology was used to determine the distribution of bacterial communities on the biological packing materials from different zones of the ABR, and hence their relationship to pollutants removal.

## Materials and methods

### Wastewater and inoculated sludge

Wastewater was collected from an ASP flooding water treatment station in the Daqing oil field (Heilongjiang Province, China) using plastic buckets (50 L). Ranges for the typical chemical composition and associated parameters in the wastewater are given in Table 1. The inoculated activated sludge was obtained from the secondary sedimentation tank at the Chengfengzhuang wastewater treatment plant (capacity 30000 m^3^/d; Daqing City, China). The sludge was cleaned to remove impurities prior to use as an inoculated sludge with a mixed liquor suspended solid content of 3600 mg/L.

**Table 1:** Typical ranges for the chemical composition and related parameters in wastewater influent obtained from the ASP flooding water treatment station (Daqing, China).

Parameters	Lower limit	Upper limit	Mean value
**HPAM, mg/L**	663.6	902.4	765.9
**Viscocity, mPa.s**	2.896	3.792	3.231
**Surfactant, mg/L**	190.8	267.1	233.0
**Total alkalinity, mg/L**	7786	7843	7814
**COD**	2597.3	3294.0	2985.5
**Petroleum substances, mg/L**	1055.1	1567.0	1313.7
**Total suspended solids, mg/L**	132	198	172
**Temperature, °C**	20	33	27
**pH**	10.5	10.9	10.7

### ABR setup and the operation

**Fig 1:**
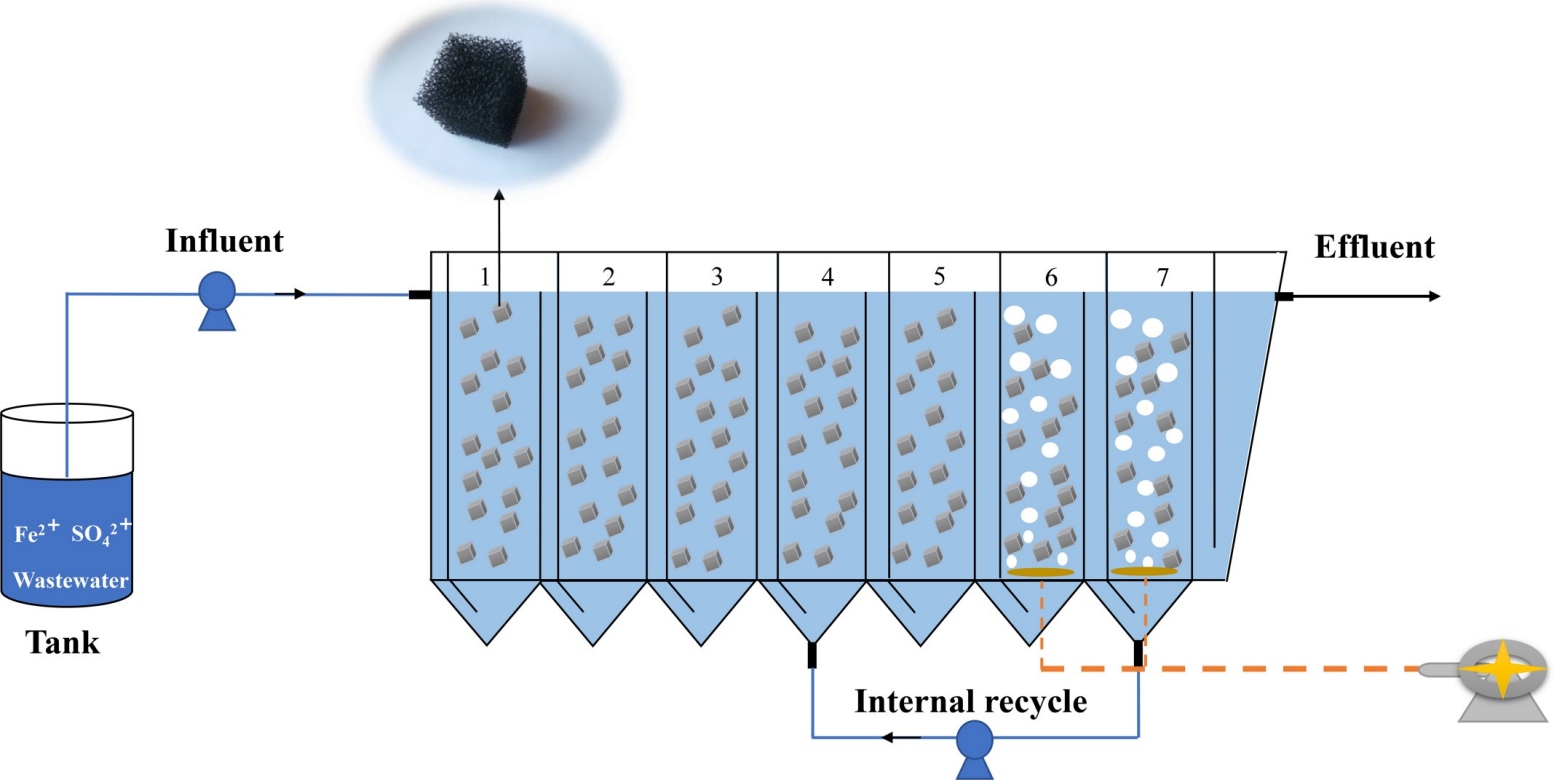
Schematic of the ABR biological reactor used for remediation of the ASP flooding process water. anaerobic zones (1–3); anoxic zones (4, 5); and aerobic zones (6, 7).

The test device was adapted from a conventional ABR and a detailed schematic is shown in Fig 1. The reactor was divided equally into seven reaction zones (labelled 1–7 in Fig 1) of 4.4 L each (30.8 L total volume). Zones labelled 1–3 were anaerobic; zones labelled 4 and 5 were anoxic; and zones labelled 6 and 7 were aerobic. The top of reaction zones 1–5 were sealed to provide a low dissolved oxygen environment for anaerobic and anoxic microorganisms. The top of reaction zones 6 and 7 were open, and continuous aeration devices were placed at the base of each zone to maintain the dissolved oxygen content of the water. All reaction zones were filled with cubes (2 cm × 2 cm) of polyurethane biological packing material. The filling capacity of reaction zones 1–5 was 50%, while that of zones 6 and 7 was 30%.

A continuous flow system was used to lift the wastewater to the unit with a BT100-2J peristaltic pump (Lan Ge, Hebei, China). The hydraulic retention time of the reactor was 48 h. The effluent from the aerobic reaction zone labelled as 7 in Fig 1 flowed back to the bottom of the first anoxic reaction zone (Labelled 4 in Fig 1) via a peristaltic pump. The reflux ratio was maintained at 1:1. In the start-up stage, activated sludge (seed) was poured into each reaction zone (1/10 of the volume) and the reactor was maintainded at ambient temperature (20–33°C) for 90 d. Samples were collected from the following locations for testing every 3 d: influent; the anaerobic, anoxic and aerobic reaction zones; and the effluent.

### Physico-chemical analysis of test samples

Test samples (influent, effluent and each zone of the ABR) were collected every 3 d using 50 mL sterile syringes and transferred into 330 mL sample bottles for analysis. The content of polymers and surfactants were determined by spectrophotometry and titration respectively according to the oil and gas industry standard method SY/T 5329–12 (Oil and gas development Standardization Technical Committee, 2012). Viscosity was determined using an AR1500ex rheometer (TA Instruments, New Castle, DE, USA). Petroleum substances in wastewater were extracted with petroleum ether, and then measured by colorimetry at 430 nm with a DR5000 (HACH, USA). Suspended solids were determined with a JBFT-03 suspended solids analyzer (Harbin Jingbo, China); the mass of solid material (g) recovered with a 0.45 μm membrane filter was used to calculate the amount of suspended solids in a measured volume (L) of wastewater.COD testing was carried out according to the American Public Health Association standard method [27]. A multi 3420 multi-parameter meter (WTW, Weilheim, Germany) was used to monitor pH and temperature. The degradation of organic matter was investigated by the analysis of organic chemicals in the wastewater using a 7890A/5975C GC-MS system (Agilent Technologies Agilent Technologies, Inc. Shanghai, China).

### High throughput DNA sequencing

After 90 d of stable operation, test samples were collected from the biological packing materials in the anaerobic (A-1, A-2, A-3), anoxic (B1, B-2) and aerobic reaction zones (C-1, C-2) of the ABR. The biofilms on the packing materials were washed and collected under sterile conditions. A MO BIO PowerSoil™ DNA isolation kit (Qiagen, Hilden, Germany) was used for DNA extraction according to the method of Wan et al. [28]. The concentration and purity of the extracted DNA was determined using a 2000c UV-Vis spectrophotometer (Thermo Scientific, USA). Samples (5μL) were separated by 1% agarose gel electrophoresis to isolate the genomic DNA. Pure samples were then amplified by the polymerase chain reaction (PCR) method on an ABI GeneAmp 9700 (Thermo Scientific, USA). Conservative primers 338F (5′-ACTCCTACGGAGGCAGA-3′) and 806R (5′- GGACTACHVGGGGTWTCTA-AT-3′) were used for PCR amplification. The PCR amplification process was performed using the TransStart® FastPfu DNA Polymerase kit based on the procedure of Huang et al. [29]. The amplified products were purified and sequenced by Shanghai Majorbio Bio-Pharm Technology Co., Ltd. (Shanghai, China). The 16S rRNA gene sequences were compared with the NCBI BLAST® database, and then analyzed according to the sequence analysis method of Hao et al. [30].

## Results and discussions

### The efficiency of the ABR for the treatment of ASP flooding wastewater

#### Pollutants removal efficiency

**Fig 2:**
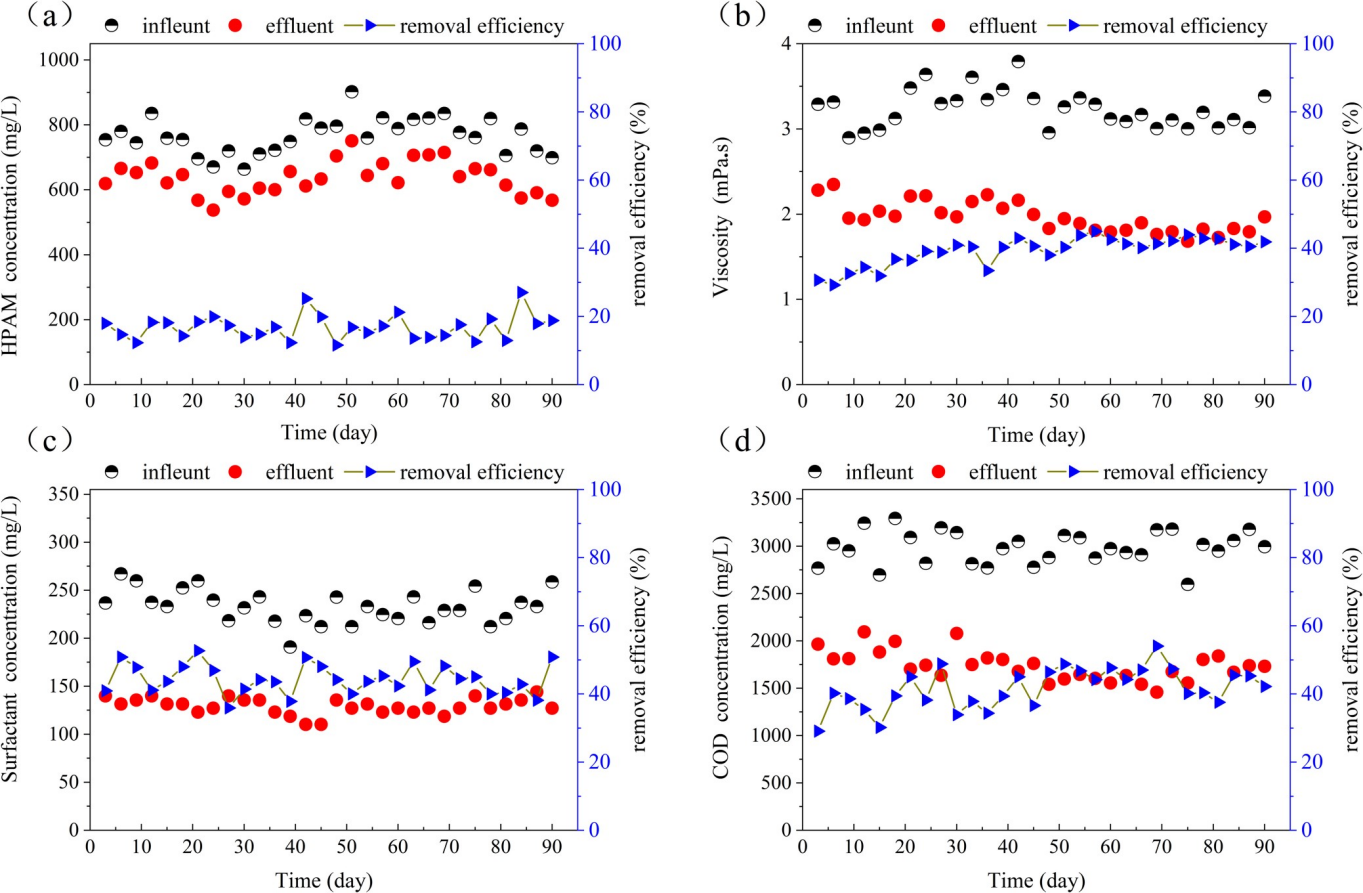
Treatment efficiency of the modified ABR reactor for the remediation of ASP flooding wastewater. (a) HPAM removal; (b) viscosity change; (c) surfactants removal; and (d) COD change.

Compared with treated water, the presence of residual polymers, alkaline surfactants, inorganic salts and other chemicals in ASP flooding wastewater shows the characteristics of increased viscosity, a higher degree of emulsification and greater stability. These characteristics increase the complexity of wastewater treatment [31]. The typical pollutants in wastewater from ASP flooding are mainly determined by the strong alkali used and include polymers, surfactants and petroleum products, etc. The variations of these pollutants together with the associated changes in viscosity and COD in the ABR treated wastewater during the test period are shown in Fig 2.

**Fig 3:**
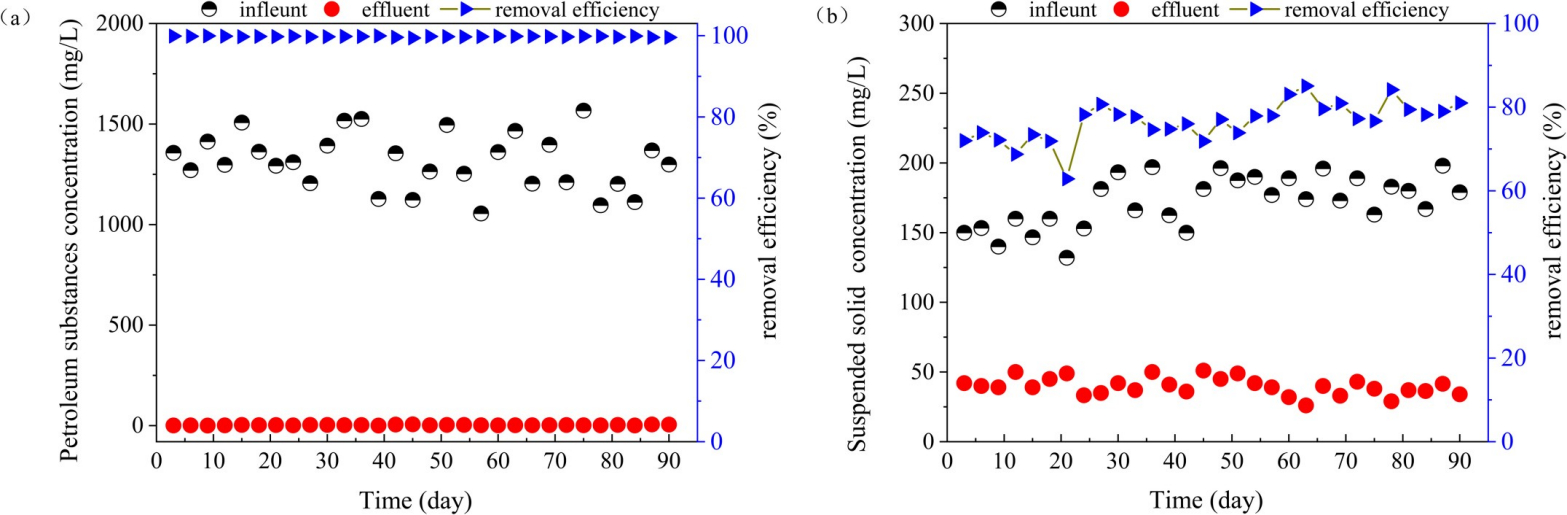
Removal efficiency of the ABR system for (a) Petroleum substances; and (b) suspended solids.

All measured parameters showed fluctuations in the influent/effluent values which reflected the typical variability of the commercial process water used in this work. For example, HPAM in the influent ranged from 663.6 to 902.4 mg/L (mean 765.9 mg/L) while the polymer contents in the effluent ranged from 571.6 to 750.7 mg/L (mean 637.1 mg/L). This equated to a mean polymer removal efficiency of 16.79% (Fig 2(A)). According to previous studies, these degradation characteristics could be related to the composition and pH of the influent (Yan et al., 2016). At high concentrations of residual petroleum substances in the influent, the hydrocarbons were preferentially consumed by microbial activity compared with high molecular weight polymers. This was consistent with the high concentration of petroleum substances in the wastewater influent and their efficient degradation (99%; Fig 3A). Furthermore, the pH of the influent water (10.7) was much higher than optimum values for most microorganisms (7.5–8.0). This could result in decreased microbial growth and metabolic activity, and hence reduced polymer treatment efficiency.

Fig 2(B) shows that the influent viscosity ranged from 2.896 to 3.792 mPa.s (mean 3.231 mPa.s) while the corresponding effluent viscosity ranged from 1.682 to 2.348 mPa.s (mean 1.963 mPa.s). The mean decrease in wastewater viscosity during the test period was 39.20%. Zhao et al. [32] demonstrated that the viscosity of a polymer solution was positively correlated with HPAM. Hence, the decrease of wastewater viscosity could be attributed to the degradation of HPAM by microorganisms (mean decrease 16.79%; Fig 2(A). Studies have shown that microorganisms can degrade macromolecular HPAM into smaller degradation products. A mechanism based on the hydrolysis of the amide side group of HPAM by bacterial amidase and their partial replacement with hydroxyl groups was proposed [33].

The hydrophilic and hydrophobic functional groups of surfactants play an important role in the stability of ASP flooding wastewater. Surfactants can reduce the interfacial tension and zeta potential, which gives ASP flooding waste stable emulsification characteristics. The variation of surfactant content in wastewater during the test period is shown in Fig 2(C). The influent surfactant concentration ranged from 190.8 to 267.1 mg/L (mean 233.0 mg/L) while the corresponding effluent ranged from 110.2 to 144.2 mg/L (mean 129.3mg/L). The average removal rate was 44.3% in the whole test period.This decrease in the wastewater surfactant content reduced its overall stability, making it more conducive to subsequent oil and water separation.

Fig 2(D) shows the change in COD of wastewater during the test cycle. The influent COD fluctuated from 2597.3 to 3294.0 mg/L (mean 2985.5 mg/L) and the effluent COD ranged from 1459.1 to 2094.6 mg/L (mean 1737.7 mg/L), representing a mean treatment efficiency was 41.6%. The significant decrease of COD in the wastewater was probably due to the preferential removal of petroleum substances (Fig 3(A)) during biodegradation.

Hence these results demonstrated that the ABR could decrease the stability and viscosity of the ASP flooding wastewater by reducing its polymer and surfactant contents.

#### Evaluation of treated ASP flooding wastewater for reuse

To preserve clean water resources and maximize recycling, ASP flooding wastewater can be used as reinjection water after appropriate treatment [34]. According to the national standard relating to the reinjection water (SY/T 5329–2012; Oil and gas development Standardization Technical Committee, 2012), the contents of oil and suspended solids in the wastewater were selected as the evaluation indexes for the wastewater post-remediation.

Fig 3(A) shows that the treatment efficiency of petroleum substances in the ASP flooding process water using ARB was relatively stable during the test period. The content of petroleum substances in the influent ranged from 1055.1 to 1567.0 mg/L (mean 1313.7 mg/L), the corresponding content of petroleum substances in the effluent ranged from 0.5 to 6.2 mg/L and the average mean removal efficiency was 99.8%. The mean content of petroleum substances during the test period (2.8 mg/L) was significantly less than the limit set by the national standard (SY/T 5329–2012; 5 mg/L). During the test period, the content of suspended solids in the influent ranged from 132 to 198 mg/L (mean 172 mg/L) and 26–51 mg/L in the treated effluent, corresponding to a mean removal efficiency of 77%. The mean suspended solids content of the effluent was 40 mg/L, which did not meet the requirements of SY/T 5329–2012 (40 mg/L). To achieve the reinjection requirement, it may be necessary to install a simple filtration device post ABR treatment, such as the pressure filtration processes widely used in oilfield wastewater treatment stations.

### Degradation of pollutants in different zones of the ABR

**Fig 4:**
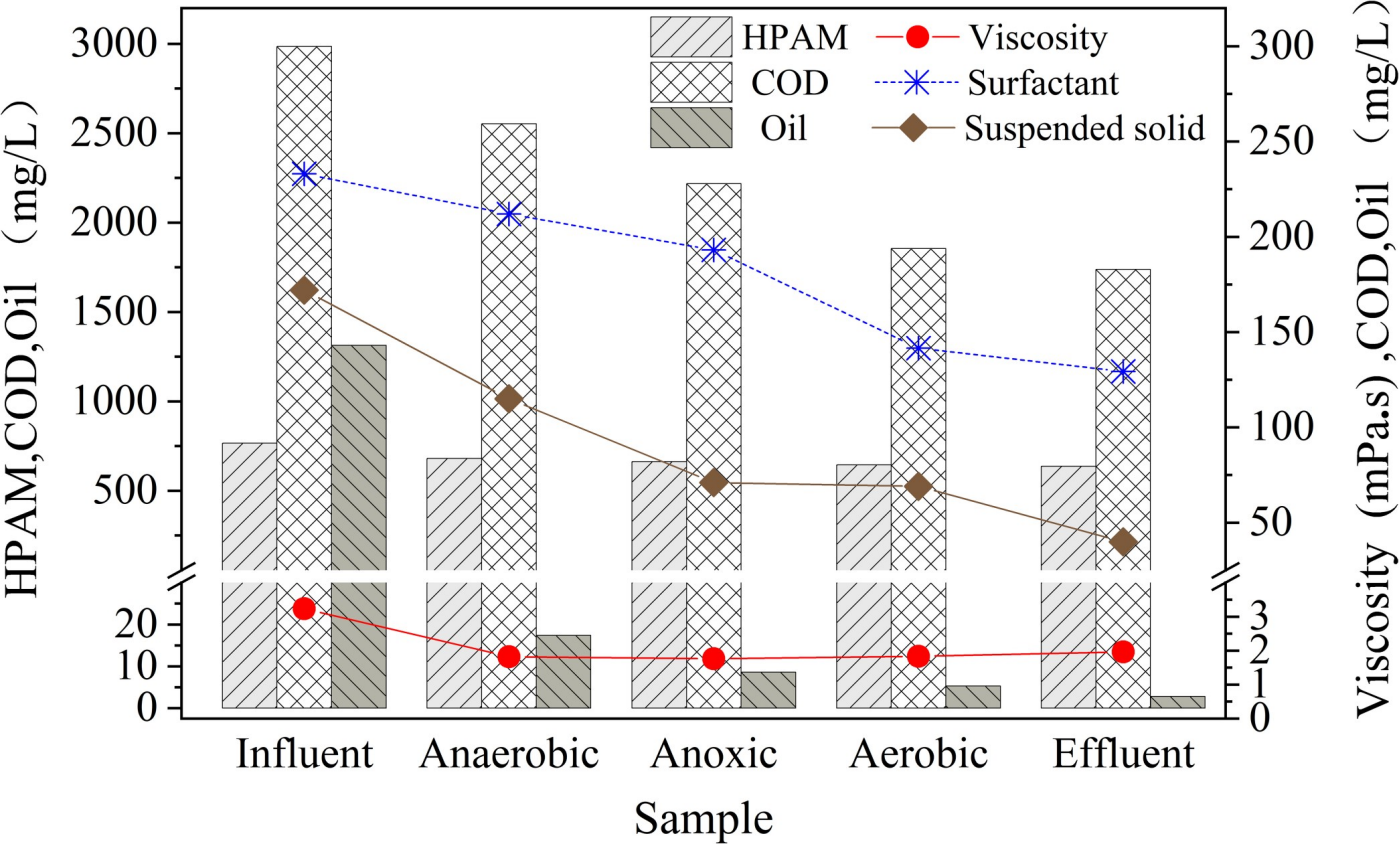
Mean values (90 d) of pollutants and associated parameters in different zones of the ABR.

The combined processes of anaerobic (three stages), anoxic (two stages) and aerobic (two stages) biodegradation were used in the ABR. To further understand the mechanisms of pollutants removal in the system, the concentrations of each contaminant and their associated parameters were determined in each zone of the ABR. The mean values of HPAM, viscosity, surfactant, COD, oil and suspended solids from the 90 d test, measured in different zones of the ABR, are shown in Fig 4.

The removal efficiencies of HPAM were 11.19%, 2.54% and 2.78% in the anaerobic, anoxic and aerobic sections, respectively indicating that the hydrolysis of HPAM mainly occurs in the anaerobic zone of the reactor. Fig 4 also shows that the mean viscosity of the reactor effluent was lower than that of the influent, consistent with the degradation of HPAM in the wastewater. However, the viscosities of the aerobic reaction zones and final effluent were higher than that of anaerobic and anoxic reaction zones (but lower than that of influent). We speculated that extracellular polymers produced by some microorganisms in the aerobic zone could fuse with the polymeric material in the wastewater, thereby increasing its viscosity.

The removal efficiencies of the surfactants were 9.01%, 8.92% and 26.67% in the anaerobic, anoxic and aerobic reaction zones, respectively, again indicating that most of the pollutant was removed during the aerobic process.

The mean reduction efficiencies in COD were 14.5%, 13.1% and 16.4% in the anaerobic, anoxic and aerobic zones respectively. It can be seen from Fig 4 that the mean concentration of petroleum substances in the influent (1313.7 mg/L) reduced to 17.4 mg/L in the anaerobic zone representing a high removal efficiency (98.7%). The removal efficiencies in the anoxic and aerobic processes were 50.5% and 38.4%, respectively.

The removal efficiencies of suspended solids were 33.1%, 38.3% and 2.82% in the anaerobic, anoxic and aerobic zones respectively, indicating that removal of the suspended material was relatively low in the aerobic process. This phenomenon may be due to continuous aeration in aerobic process, and the biofilm shedding and sedimentation at the bottom of the reactor are stirred up, resulting in an increase in suspended solids content in the final effluent and a decrease in removal rate.

### Evolution of organic compounds in each zone of the ABR

GC-MS was used to study the evolution of organic compounds resulting from the biodegradation of pollutants in each zone of the ABR. Details of the specific organic compounds monitored in each zone are given in S1 Table. S1 Fig. shows the GC-MS total ion chromatograms (TIC) obtained for samples taken from each region of the ABR, and the increase in the intensity of the TIC at the first anaerobic zone (labelled AN1). The results given in S1 Table show that some influent organic compounds were degraded after AN1 into a range of smaller chemicals. Combined with the observed reductions in petroleum substances and COD (Fig 4), these results also confirmed the preferential degradation and utilization of petroleum substances by microorganisms in the anaerobic zone. However, it can be seen from S1 Fig and S1 Table that some organic compounds remained following subsequent treatments in each zone, demonstrating that some pollutants could not be degraded by biological treatment.

The changes of main types of organic matter in each reaction zone are as shown in Table 2. The main organic compounds that could be determined by GC-MS in the influent were n-alkanes (C_11-31_), branched alkanes, aromatic compounds, esters, alcohols, ketones and cyclosiloxanes. After treatment in the anaerobic region (zones 1–3), the main organic pollutants detected in the third zone were n-alkanes (C_7-20_), branched alkanes, esters, silanes and siloxanes. After treatment in the anoxic region (zone 4, 5), the organic pollutants detected in the zone five were n-alkanes (C_7-12_), branched alkanes, siloxanes and esters. The organic compounds detected in the effluent of zone seven of the aerobic region (zones 6, 7) were n-alkanes (C_10_), branched alkanes, cycloalkanes and siloxanes.

**Table 2:** Main organic matter types in each zone.

Organic compounds	Influent	AN1	AN2	AN3	AN4	AN5	O1	O2
Alkanes	+	+	+	+	+	+	+	+
Aromatic hydrocarbons	+	+	-	-	-	-	-	-
Esters	+	-	+	+	+	+	-	+
Alcohol	+	-	-	-	-	-	-	-
Ketone	+	-	-	-	-	-	-	-
Silane	-	-	-	+	-	-	-	-
Siloxane	+	+	+	+	+	+	+	+

The n-alkanes in the influent ranged from C_11_to C_31_. After treatment in the anaerobic, anoxic and aerobic reaction zones, the length of the alkane carbon chain gradually decreased. Cyclosiloxane, present in the influent, was detected in all reaction zones, while siloxane and silane were found in the anaerobic, anoxic and aerobic zones. These results suggested that microorganisms had a limited capacity to degrade cyclosiloxane during the treatment process. Aromatic hydrocarbons, esters, alcohols, ketones, and some normal and branched chain alkanes, present in the influent, were not detected in the effluent. Hence these organic compounds were biodegradable and could be removed from the ASP flooding wastewater by the ABR reactor.

### Bacterial diversity in the ABR

#### Biodiversity and abundance analysis

S2 Table shows the total number of sequencing reads (55014) available representing operational taxonomic units (OTU) in samples obtained from each zone of the ABR. The coverage rate for the samples (> 99.3%) indicated that a high proportion of the reads could be explained by the OTUs.

S2(A) Fig shows the dilution curves representing the number of sequences randomly selected from the samples versus the number of reads. At ~ 20,000 reads the curves for each sample approximated to linear indicating that the sample sequencing data was reasonable. The comparable values obtained for each dataset from the Shannon-Wiener curve (S2(B) Fig) suggested that the sequencing data could be used to obtain microbial information about the samples.

#### Differential analysis of bacterial populations

S3 Fig shows the OTU coincidence Venn diagrams for the samples from different zones of the ABR. The OTU coincidence for the anaerobic, anoxic and aerobic samples were 466, 623 and 464, respectively. The OTU coincidence between A-1 and A-2, A-1 and A-3, A-2 and A-3 were 520, 607 and 565, respectively. Although the degree of coincidence between the first and third zones of the anaerobic region (607) was a little higher, the degree coincidence of the two anoxic reaction zones (623) was the highest.

#### Distribution and comparison of bacteria in different regions of the ABR

**Fig 5:**
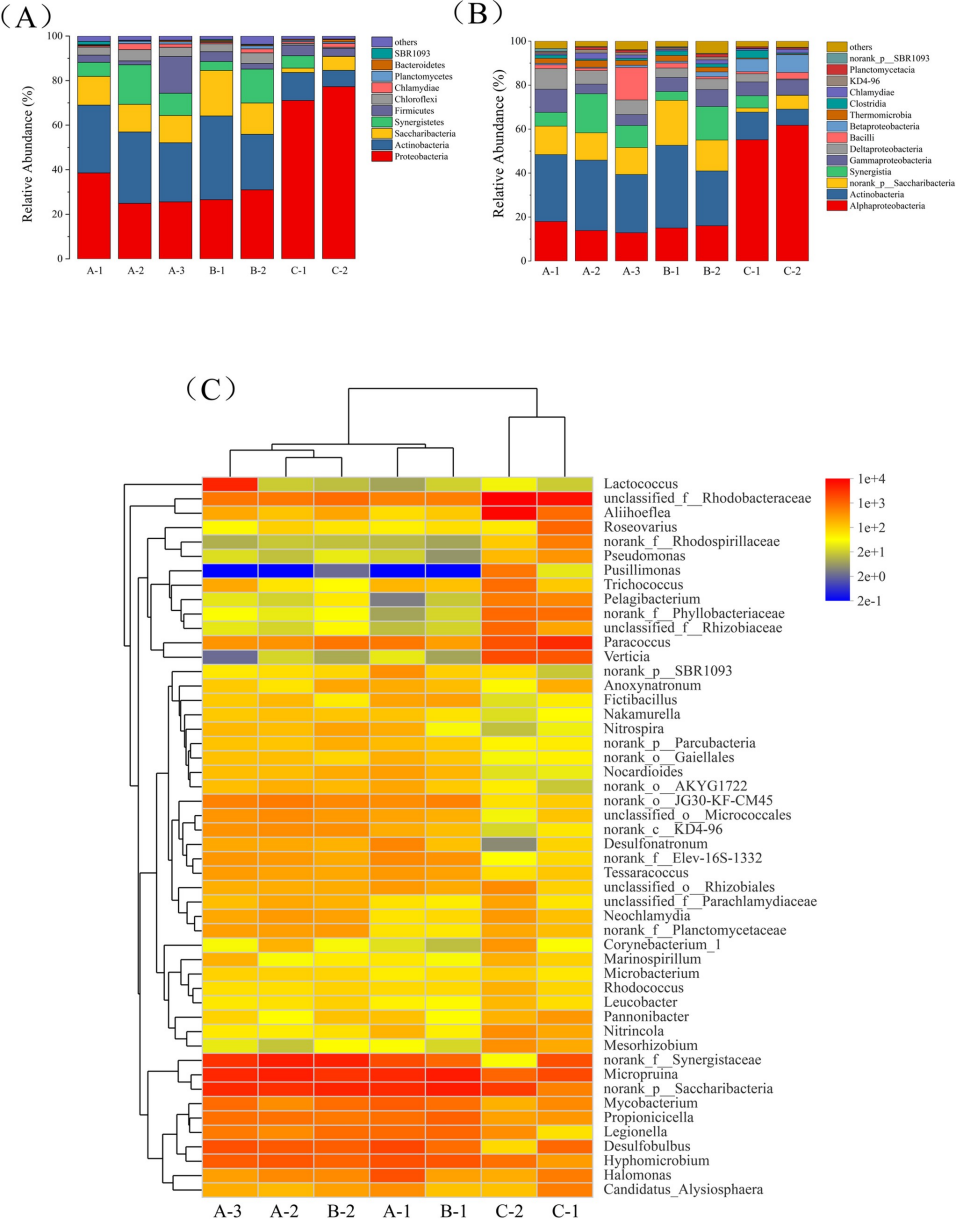
Distribution and comparison of bacteria from each zone of the ABR (communities < 1% are denoted as others). (A) Phyla distribution; (B) class distribution; and (C) genus distribution.

To study the composition of the bacterial communities in the different zones of the ABR, species were characterized at the level of phylum, class and genus (Fig 5).The phyla distribution and comparison of bacteria in each zone of the ABR is shown in Fig 5(A). The diversity of bacteria in the anaerobic and anoxic zones was relatively small and comprised mainly *Proteobacteria*, *Actinobacteria*, *Saccharibacteria*, *Synergistetes*, *Firmicutes* and *Chloroflexi*. Most microorganisms in A-1 were the *Proteobacteria* (38.6%) and *Actinobacteria*, with lesser amounts in A-2 and A-3; the populations of *Synergistetes* (17.7%) and *Firmicutes* (16.5%) showed some growth in zones A-2 and A-3 respectively. Compared with the anaerobic zones, the populations of *Actinobacteria* (B-1 = 37.6%, B-2 = 24.9%) and *Saccharibacteria* (B-1 = 20.4%, B-2 = 14.1%) increased in the anoxic stage. The three main phyla in the two aerobic zones were the *Proteobacteria* (B-1 = 71.1%, B-2 = 77.3%), *Actinobacteria* (B-1 = 12.6%, B-2 = 7.4%), and *Firmicutes* (B-1 = 4.8%, B-2 = 3.5%). Apart from the *Proteobacteria*, which increased markedly, the populations of all other microbes decreased relative to the anaerobic and anoxic zones. The dominant bacteria in the seven zones of the ABR were the *Proteobacteria*. The *Proteobacteria*, together with *Actinobacteria* and *Chloroflexi*, are the major microbial communities in wastewater treatment plants [35]. *Proteobacteria*, *Firmicutes* and *Actinobacteria* have the ability to degrade hydrocarbons [36–38], and these are important classes of microorganisms that degrade petroleum hydrocarbons. *Synergistetes* were found to be the main community of oil hydrocarbon degrading bacteria in anaerobic digestion [39]. *Proteobacteria* and *Firmicutes* have been shown to hydrolyze HPAM into smaller molecular weight oligomers [40]. *Chloroflexi* is also a key microorganism for HPAM degradation [41].

The class distribution and comparison of bacteria in each zone of the ABR is shown in Fig 5(B). The main classes of bacteria in anaerobic zones were: *Actinobacteria* (A-1 = 30.4%, A-2 = 32.0%); *Alphaproteobacteria* (A-1 = 18.0%, A-2 = 13.9%); *Saccharibacteria* (A-1 = 12.9%, A-2 = 12.4%); *Gammaproteobacteria* (A-1 = 10.5%, A-2 = 4.4%); *Deltaproteobacteria* (A-1 = 9.4%, A–2 = 6.2%); and *Synergistia* (A-1 = 6.3%, A-2 = 17.7%). The *Bacilli* showed a significant increase in A-3. The distribution of microbial species in the anoxic zones and anaerobic zones were similar although the population of *Actinobacteria* (B-1 = 37.6%, B-2 = 24.9%) and *Saccharibacteria* (B-1 = 20.4%, B-2 = 14.1%) were slightly higher. Compared with the pevious zones, the populations of *Alphaproteobacteria* (C-1 = 55.2%, C-2 = 61.8%) and *Betaproteobacteria* (C-1 = 5.8%, C-2 = 8.1%) were significantly higher in C-1 and C-2, while amounts of most other microbes, in particular *Actinobacteria*, *Synergistia* and *Saccharibacteria* were reduced. The main populations of microrganisms in the aerobic zones comprised the following classes: *Alphaproteobacteria* (C-1 = 55.2%, C-2 = 61.8%), *Actinobacteria* (C-1 = 12.6%, C-2 = 7.4%), *Gammaproteobacteria* (C-1 = 6.3%, C-2 = 7.1%) and *Betaproteobacteria* (C-1 = 5.8%, C-2 = 8.1%). The populations *Actinobacteria* were higher in the anaerobic and anoxic reaction zones, while the *Alphaproteobacteria* were predominant in the aerobic zones. Studies have shown that *Alpha-*, *Gamma-* and *Deltaproteobacteria* (phylum *Proteobacteria)* may play an important role in the degradation of hydrocarbons [42].

The genus distribution and comparison of bacteria from each zone of the ABR is given in Fig 5(C). The main bacteria in A-1 were the genus *Micropruina* (13.4%) and phylum *Saccharibacteria* (12.9%). A-2 comprised mainly genus *Micropruina* (17.9%), family *Synergistaceae* (17.6%) and phylum *Sacharibacteria* (12.4%). The main bacteria in A-3 were the genera *Lactococcus* (13.5%) and *Micropruina* (12.9%) together with *Saccharibacteria* (12.2%) and *Synergistaceae* (9.9%). B-1 comprised mainly the genus *Micropruina* (21.2%) and the *Saccharibacteria* (20.4%). The populations of *Synergistaceae* (15.1%) increased in B-2, while the *Micropruina* (10.1%) and *Saccharibacteria* (14.1%) decreased. Microorganisms of family *Rhodobacteraceae* (C-1 = 21.7%, C-2 = 22.1%) predominated in the aerobic zones. The genera *Paracocus* (12.2%) and *Aliihoeflea* (19.8%) were higher in C-1 and C-2, respectively.

*Micropruina*, *Synergistaceae*, *Saccharibacteria* and *Lactococcus* were predominant in the anaerobic reaction zones, which showed good efficiency for the reduction of COD, petroleum substances and polymers in wastewater. These microbes may also play a major role in the removal of pollutants in the anoxic reaction zones. In previous studies, it was found that Micropruina has the ability to degrade petrochemical products [43]. *Saccharibacteria* has been found to prefer complex organic compounds and refractory contaminants [44]. *Synergistaceae* in oil reservoir was found to carry the genes for the initial activation of hydrocarbons and further dearomatization of aromatic hydrocarbons [45]. *Lactococcus* plays an important role in petroleum degradation and was reported to have high efficient capacity of PAHs degradation [46,47]. *Rhodobacteraceae* had the highest microbial content in the aerobic reaction zone, which also showed good efficiency for the reduction of COD, surfactants and suspended solids. *Rhodobacteraceae* is a key participant in petroleum degradation and plays a role in the treatment of highly concentrated polycyclic aromatic hydrocarbons [48,49]. *Paracoccus* can produce biosurfactants, improve the solubility of hydrophobic compounds and enhance the degradation of petroleum [50]. PAliihoeflea can utilize aromatic hydrocarbons aerobically [51,52].

## Conclusions

An improved multi-zone anaerobic baffled reactor (ABR) using FeSO_4_ as an electron acceptor was fabricated to treat wastewater from crude oil extraction using ASP flooding technology. Unter test, the mean treatment efficiencies of the ABR for HPAM, viscosity, surfactant, COD, petroleum substances and suspended solids are 16.8%, 39.2%, 44.3%, 41.6%, 98.8% and 77.0%, respectively. In addition, the content of petroleum substances in the wastewater treated by the ABR could meet the requirements of a national standard (≤ 5 mg/L) for reinjection of the water at the Daqing Oilfield in China. However, suspended solids required additional treatment to meet the reinjection standard using technology available at oilfield treatment stations. Analysis of pollutants and associated parameters in each zone of the ABR revealed a sequential degradation process with reduction of HPAM, viscosity and petroleum substances occurring mainly in the anaerobic zones. Monitoring of organic compounds in the wastewater from each zone of the ABR showed that aromatic compounds, esters, alcohols, ketones, some n-alkanes and branched alkanes present in the influent were continuously degraded during the process. An investigation of the relationship between the bacterial communities (using DNA sequencing) and pollutant degradation in each zone of the ABR showed that *Micropruina*, *Saccharibacteria* and *Synergistaceae* were the dominant bacteria in the anaerobic and anoxic process contributing to the reductions in COD, petroleum substances and polymers in wastewater. The *Rhodobacteraceae* and *Aliihoeflea* were predominant in the aerobic zones. The improved ABR reactor showed good efficiency and stability for the treatment of ASP flooding wastewater. The results from this study may provide a basis for the development of biodegradation methods to treat wastewater from ASP flooding technology.

## Supporting information

S1 Fig GC-MS TIC of samples taken from each region of the ABR reactor (AN1-AN3 = anaerobic zones; AN4, AN5 = anoxic zones; O1, O2 = aerobic zones). (TIF)

S2 Fig Dilution curves for samples taken from each zone of the ABR (A-1, A-2, A-3 = anaerobic; B-1, B-2 = anoxic; C-1, C-2 = aerobic): (a) Dilution curve; (b) Shannon-Wiener curve.
(TIF)

S3 Fig Venn diagram for showing the OTU coincidence for samples from different zones of the ABR: (A) Anaerobic: (B) anoxic (C) aerobic.
(TIF)

S1 Table Organic compounds were identified by GC-MS in samples from each region of the ABR. (DOCX)

S2 Table Number of sequences analyzed, OTU richness (Chao, ACE), Shannon and Simpson diversity indices of bacterial communities from each zone of the ABR (A-1, A-2, A-3 = anaerobic; B-1, B-2 = anoxic; C-1, C-2 = aerobic). (DOCX)
