# Supplementary figures and images for: Efficiency and bacterial diversity of an improved anaerobic baffled reactor for the remediation of wastewater from alkaline-surfactant-polymer (ASP) flooding technology
Source: PLoS One. 2022 Jan 7;17(1):e0261458. doi: 10.1371/journal.pone.0261458 (PMC8741043; doi:10.1371/journal.pone.0261458)

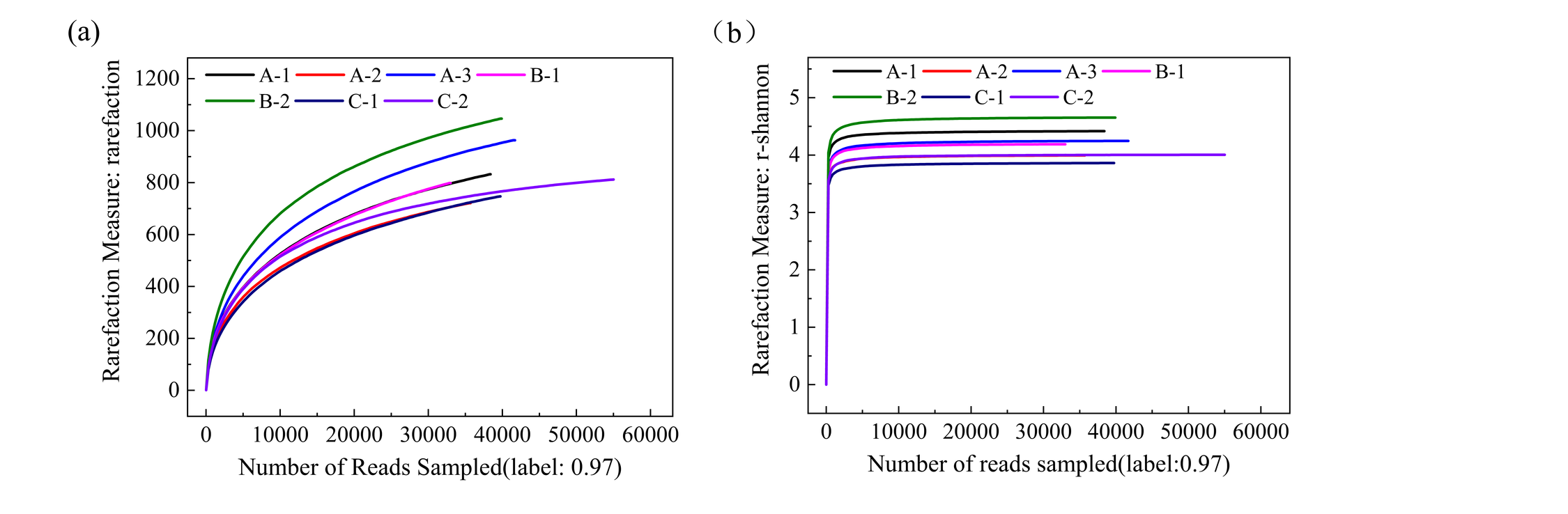

Supplement: S2 Fig — Dilution curves for samples taken from each zone of the ABR (A-1, A-2, A-3 = anaerobic; B-1, B-2 = anoxic; C-1, C-2 = aerobic): (a) Dilution curve; (b) Shannon-Wiener curve. (TIF) [file pone.0261458.s002.tif]

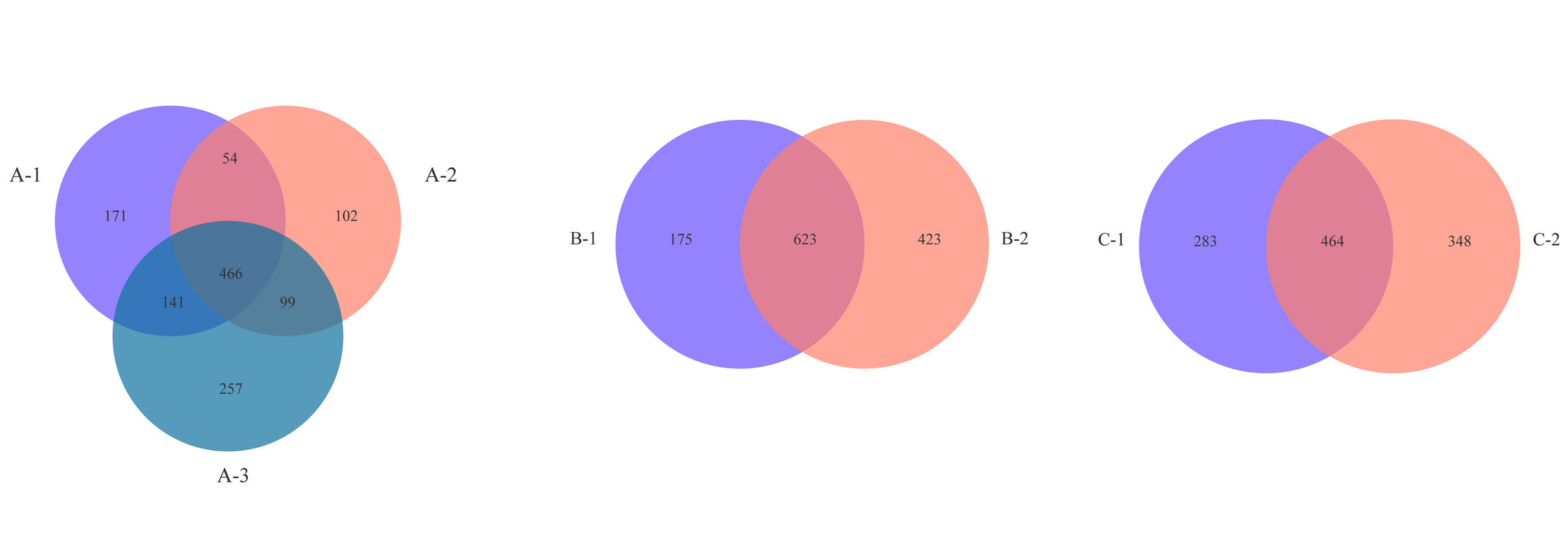

Supplement: S3 Fig — Venn diagram for showing the OTU coincidence for samples from different zones of the ABR: (A) Anaerobic: (B) anoxic (C) aerobic. (TIF) [file pone.0261458.s003.tif]
