# Supplementary material for: Efficiency and bacterial diversity of an improved anaerobic baffled reactor for the remediation of wastewater from alkaline-surfactant-polymer (ASP) flooding technology
Source: PLoS One. 2022 Jan 7;17(1):e0261458. doi: 10.1371/journal.pone.0261458 (PMC8741043; doi:10.1371/journal.pone.0261458)
Supplement: S1 Table — (DOCX) [file pone.0261458.s004.docx]

**S1 Table. Organic compounds identified by GC-MS in samples from each region of the ABR.**

| **Name** | **In** | **AN1** | **AN2** | **AN3** | **AN4** | **AN5** | **O1** | **O2** |
| --- | --- | --- | --- | --- | --- | --- | --- | --- |
| **Decane, 3,6-dimethyl-** | + | - | - | - | - | + | - | - |
| **Tetradecane, 2,6,10-trimethyl-** | + | + | - | - | - | - | - | + |
| **Undecane** | + | + | - | - | - | - | - | - |
| **trans-Decalin, 2-methyl-** | + | - | - | - | - | - | - | - |
| **1-Methyldecahydronaphthalene** | + | - | - | - | - | - | - | - |
| **Cyclohexane, pentyl-** | + | - | - | - | - | - | - | - |
| **Cyclopentasiloxane, decamethyl-** | + | + | + | + | + | + | + | + |
| **Dodecane, 2,6,10-trimethyl-** | + | - | - | - | - | - | - | - |
| **Carbonic acid, dodecyl isobutyl ester** | + | - | - | - | - | - | - | - |
| **trans,trans-1,6-Dimethylspiro[4.5]decane** | + | - | - | - | - | - | - | - |
| **6,10,13-Trimethyltetradecanol** | + | - | - | - | - | - | - | - |
| **Dodecane** | + | + | + | + | - | + | - | - |
| **Undecane, 2,6-dimethyl-** | + | - | - | - | - | - | - | - |
| **Cyclohexane, 2-butyl-1,1,3-trimethyl-** | + | - | - | - | - | - | - | - |
| **cis, cis-3-Ethylbicyclo[4.4.0]decane** | + | - | - | - | - | - | - | - |
| **Cyclohexane, (2-methylpropyl)-** | + | - | - | - | - | - | - | - |
| **2-Tetradecanol** | + | - | - | - | - | - | - | - |
| **Sulfurous acid, 2-propyl undecyl ester** | + | - | - | - | - | - | - | - |
| **Nonane, 3-methyl-** | + | - | - | - | - | - | - | - |
| **Hentriacontane** | + | + | - | - | - | - | + | - |
| **Tridecane** | + | + | - | - | - | - | - | - |
| **Sulfurous acid, nonyl 2-propyl ester** | + | - | - | - | - | - | - | - |
| **Decane, 2-cyclohexyl-** | + | - | - | - | - | - | - | - |
| **Cyclohexanone, 3-butyl-** | + | - | - | - | - | - | - | - |
| **Cyclohexane, 1-(cyclohexylmethyl)-4-methyl-, cis-** | + | - | - | - | - | - | - | - |
| **2-Dodecanol** | + | - | - | - | - | - | - | - |
| **Tetradecane** | + | + | - | - | - | - | - | - |
| **Spiro[5.6]dodecane-1,7-dione** | + | - | - | - | - | - | - | - |
| **trans, cis-3-Ethylbicyclo[4.4.0]decane** | + | - | - | - | - | - | - | - |
| **Dodecane, 2-cyclohexyl-** | + | - | - | - | - | - | - | - |
| **Hexadecane** | + | + | - | - | - | - | - | - |
| **Decahydro-4,4,8,9,10-pentamethylnaphthalene** | + | + | - | - | - | - | - | - |
| **trans-anti-trans-Tetra-decahydroanthracene** | + | - | - | - | - | - | - | - |
| **Octadecane, 3-ethyl-5-(2-ethylbutyl)-** | + | - | - | - | - | - | - | - |
| **Pentadecane** | + | - | - | - | - | - | - | - |
| **Bicyclo[3.1.1]heptan-2-one, 6,6-dimethyl-, (1R)-** | + | - | - | - | - | - | - | - |
| **Tetrapentacontane, 1,54-dibromo-** | + | - | - | - | - | - | - | - |
| **3,7-Dimethyl-6-nonen-1-ol acetate** | + | - | - | - | - | - | - | - |
| **Tridecane, 3-methyl-** | + | - | - | - | - | - | - | - |
| **Undecane, 3-cyclohexyl-** | + | + | - | - | - | - | - | - |
| **1H-Pyrazole-1-carboxaldehyde, 4-ethyl-4,5-dihydro-5-propyl-** | + | - | - | - | - | - | - | - |
| **Heptadecane** | + | + | - | - | - | - | - | - |
| **Tridecane, 4-cyclohexyl-** | + | - | - | - | - | - | - | - |
| **Octadecane** | + | + | - | - | - | - | - | - |
| **2-Piperidinone, N-[4-bromo-n-butyl]-** | + | - | - | - | - | - | - | - |
| **Heptacosane** | + | - | - | + | - | + | - | - |
| **Eicosane** | + | + | - | + | - | - | + | - |
| **Sulfurous acid, cyclohexylmethyl octadecyl ester** | + | - | - | + | - | - | - | - |
| **Heneicosane** | + | - | - | - | - | - | - | - |
| **Tricosane** | + | + | - | - | - | - | - | - |
| **2-Methyl-7-phenylindole** | + | - | - | - | - | - | - | - |
| **Cyclotetrasiloxane, octamethyl-** | - | + | - | - | - | + | - | - |
| **Tridecanal** | - | + | - | - | - | - | - | - |
| **Undecane, 3,7-dimethyl-** | - | + | - | - | - | - | - | - |
| **Octatriacontyl trifluoroacetate** | - | + | - | - | - | - | - | - |
| **cis-Decalin, 2-syn-methyl-** | - | + | - | - | - | - | - | - |
| **Hexadecane, 3-methyl-** | - | + | - | - | - | - | - | - |
| **Tetradecane, 4-ethyl-** | - | + | - | - | - | - | - | - |
| **Tridecanol, 2-ethyl-2-methyl-** | - | + | - | - | - | - | - | - |
| **Cyclohexasiloxane, dodecamethyl-** | - | + | + | + | + | + | + | + |
| **Cyclohexane, 1,1'-(1,3-propanediyl)bis-** | - | + | - | - | - | - | - | - |
| **1,1-Dimethyl-1-silacyclo-3-pentene** | - | + | - | - | - | - | - | - |
| **3-Cyclobut-1-enyl-3-hydroxy-2-methyl-propionic acid** | - | + | - | - | - | - | - | - |
| **Methoxyacetic acid, 2-tetradecyl ester** | - | + | - | - | - | - | - | - |
| **Cyclopentanecarboxylic acid, 4-hexadecyl ester** | - | + | - | - | - | - | - | - |
| **Nonadecane** | - | + | - | - | - | - | - | - |
| **Heptadecane, 2,6,10,15-tetramethyl-** | - | + | - | - | - | - | - | - |
| **Benzo[h]quinoline, 2,4-dimethyl-** | - | + | - | - | - | - | - | - |
| **Decane, 2,4,6-trimethyl-** | - | - | + | - | - | - | - | - |
| **Undecane, 5,7-dimethyl-** | - | - | + | - | - | - | - | - |
| **Decane, 2,3,7-trimethyl-** | - | - | + | - | - | - | - | - |
| **2-Thiopheneacetic acid, 2-butyl ester** | - | - | + | - | - | - | - | + |
| **Hexasiloxane, 1,1,3,3,5,5,7,7,9,9,11,11-dodecamethyl-** | - | - | + | + | - | + | + | + |
| **Silane, trichlorooctadecyl-** | - | - | - | + | - | - | - | - |
| **3-Ethyl-3-methylheptane** | - | - | - | + | + | - | - | - |
| **Octane, 3-ethyl-2,7-dimethyl-** | - | - | - | - | + | - | - | - |
| **Triacontane** | - | - | - | - | + | - | - | - |
| **2-Thiopheneacetic acid, 2-methylpropyl ester** | - | - | - | - | + | - | - | - |
| **Undecane, 2,7-dimethyl-** | - | - | - | - | - | + | - | - |
| **Sulfurous acid, cyclohexylmethyl tridecyl ester** | - | - | - | - | - | + | - | - |
| **Heneicosane, 3-methyl-** | - | - | - | - | - | - | + | - |
| **Undecane, 3,8-dimethyl-** | - | - | - | - | - | - | + | - |
| **Oxazole, 2,4-dimethyl-** | - | - | - | - | - | - | + | - |
| **Decane** | - | - | - | - | - | - | - | + |
| **Heptadecane, 8-methyl-** | - | - | - | - | - | - | - | + |

AN1-AN3 = anaerobic zoness; AN4, AN5 = anoxic zones; O1, O2 = aerobic zones
