# Supplementary material for: Efficiency and bacterial diversity of an improved anaerobic baffled reactor for the remediation of wastewater from alkaline-surfactant-polymer (ASP) flooding technology
Source: PLoS One. 2022 Jan 7;17(1):e0261458. doi: 10.1371/journal.pone.0261458 (PMC8741043; doi:10.1371/journal.pone.0261458)
Supplement: S2 Table — (DOCX) [file pone.0261458.s005.docx]

**S2 Table. Number of sequences analyzed, OTU richness (Chao, ACE), Shannon and Simpson diversity indices of bacterial communities from each zone of the ABR** (A-1, A-2, A-3 = anaerobic; B-1, B-2 = anoxic; C-1, C-2 = aerobic)

| **Sample** | **Reads** | **OTU** | **ACE** | **Chao** | **coverage** | **Shannon** | **Simpson** |
| --- | --- | --- | --- | --- | --- | --- | --- |
| **A-1** | 55014 | 171 | 1073 | 1067 | 0.993734 | 4.42 | 0.0356 |
| **A-2** | 55014 | 102 | 922 | 909 | 0.994314 | 3.99 | 0.0597 |
| **A-3** | 55014 | 257 | 1208 | 1158 | 0.993773 | 4.25 | 0.0487 |
| **B-1** | 55014 | 175 | 1079 | 1066 | 0.992294 | 4.19 | 0.0600 |
| **B-2** | 55014 | 423 | 1276 | 1298 | 0.993331 | 4.65 | 0.0333 |
| **C-1** | 55014 | 283 | 981 | 939 | 0.994401 | 3.86 | 0.0649 |
| **C-2** | 55014 | 348 | 890 | 881 | 0.997655 | 4.01 | 0.0675 |
